# First report of *Kocuria marina* spontaneous peritonitis in a child

**DOI:** 10.1186/s12879-014-0719-5

**Published:** 2014-12-30

**Authors:** Gabriel Brändle, Arnaud G L’Huillier, Noémie Wagner, Alain Gervaix, Barbara E Wildhaber, Laurence Lacroix

**Affiliations:** Pediatric Emergency Medicine, Child and Adolescent Department, University Hospitals of Geneva, Avenue de la Roseraie 47, Geneva 14, CH-1211 Switzerland; Pediatric Infectious Diseases, Child and Adolescent Department, University Hospitals of Geneva, Rue Willy Donzé 6, Geneva, 1205 Switzerland; Pediatric Surgery, Child and Adolescent Department, University Hospitals of Geneva, Rue Willy Donzé 6, Geneva, 1205 Switzerland; 37 Bd de la Cluse, Geneva, CH-1205 Switzerland; Service d’Accueil et d’Urgences Pédiatriques, Hôpitaux Universitaires des Genève, Avenue de la Roseraie 47, Geneva 14, CH-1211 Switzerland

**Keywords:** Spontaneous bacterial peritonitis, Primary peritonitis, Pediatrics, Kocuria, Kocuria marina

## Abstract

**Background:**

Spontaneous bacterial peritonitis (SBP) is a rare affection in the pediatric population. It usually occurs when concurrent conditions are present, such as nephrotic syndrome, peritoneal dialysis or liver disease. We report a case of spontaneous bacterial peritonitis due to *Kocuria marina* in a 2-year-old child with no underlying risk factor. This is both the first description of an infection caused by this rare pathogen in a child and the first reported case of primary peritonitis caused by K. marina in a patient with no predisposing condition.

**Case presentation:**

A 2 year-old boy presented to the Pediatric Emergency Department with clinical signs of peritonitis. Laparoscopic surgical exploration confirmed purulent, generalized peritonitis without perforation. Culture of the peritoneal fluid revealed the presence of *Kocuria marina*, a Gram-positive coccoid environmental bacteria. After peritoneal lavage and appropriate antibiotic treatment, the patient improved and was discharged without sequel.

**Conclusion:**

The present report illustrates the first clinical presentation of *Kocuria marina* SBP in a child with no underlying risk factor. Although never previously described in healthy patients, this pathogen may therefore be considered as a possible cause of SBP in a child. This unusual finding extends the spectrum of infectious diseases caused by *Kocuria marina* beyond the scope of the previously described susceptible population.

**Electronic supplementary material:**

The online version of this article (doi:10.1186/s12879-014-0719-5) contains supplementary material, which is available to authorized users.

## Background

Peritonitis is one of the most frequent abdominal surgical emergency, both in the pediatric and the adult population. It is defined as spontaneous bacterial peritonitis (SBP), or primary peritonitis, when a bacterial infection of the peritoneal cavity is present in the absence of contiguous source of infection [1]. It has been described that 1 to 2% of all pediatric abdominal emergencies requiring surgical intervention are due to SBP [2]. However, this condition rarely develops in previously healthy children without any underlying medical condition [3]. SBP peaks between 5 and 9 years of age, and is most often described in susceptible patients, especially those prone to ascites (i.e. nephrotic syndrome, chronic liver disease and cardiac insufficiency) or undergoing peritoneal dialysis. Hematogenous inoculation seems to be the main pathophysiologic mechanism, but peritoneal spread via the lymphatics, translocation through the intestinal wall, and ascending infection from the female genital tract have also been raised [1]. SBP in children is mainly caused by *Streptococcus pneumoniae*. However, Gram-negative enteric bacteria (especially *Escherichia coli* and *Klebsiella pneumoniae*), *Staphylococcus* species and *Streptococcus pyogenes* strains are also common isolated pathogens [4]. *Kocuria marina* is an exceedingly rare cause of SBP and it has never been encountered to be the causative agent of SBP in a previously healthy patient. Moreover, no pediatric case of SBP due to this pathogen has ever been described.

We report the first case of SBP caused by *Kocuria marina* in a child with no previous risk factors.

## Case presentation

A 2.5-year-old caucasian boy presented to the Pediatric Emergency Department with a one-day history of lethargy and two episodes of bilious vomiting during the past three hours, without any fever. The symptoms appeared two days after he had returned from a 10-day holiday in Crete (Greece), where he ate seafood and fish every day. A cefpodoxime treatment had been introduced 9 days before the admission for an upper respiratory tract infection.

His past medical history revealed mild developmental delay and poorly controlled focal epilepsy, despite valproic acid and clobazam treatment, secondary to an ischemic episode in the neonatal period. No fever or modification of the seizure pattern had been noted by the parents.

Clinical examination revealed a 15.3 kg body weight conscious but lethargic child lying still in a fetal position. Temperature was 37°C, heart rate 105/min, and blood pressure 135/88 mmHg. Palpation of the abdomen was tender and bowel sounds were absent. However, no guarding or rebound tenderness was observed. Laboratory work-up showed the following results: white blood cell count 24.8 G/L with immature neutrophils 6.2 G/L, normal CRP, procalcitonin, AST, ALT and electrolytes. Intravenous lactates were 2.3 mmol/L, and amylase and lipase were elevated (561 U/L and 1074 U/L respectively).

Abdominal ultrasound showed severe thickening of the intestinal walls in the right lower quadrant, with a large amount of multiloculated peritoneal fluid and hyperechogenic mesenteric fat. The ileocecal valve was hypervascularized. The visualized proximal part of the appendix showed no abnormality and the pancreas also had a normal aspect.

**Figure 1:**
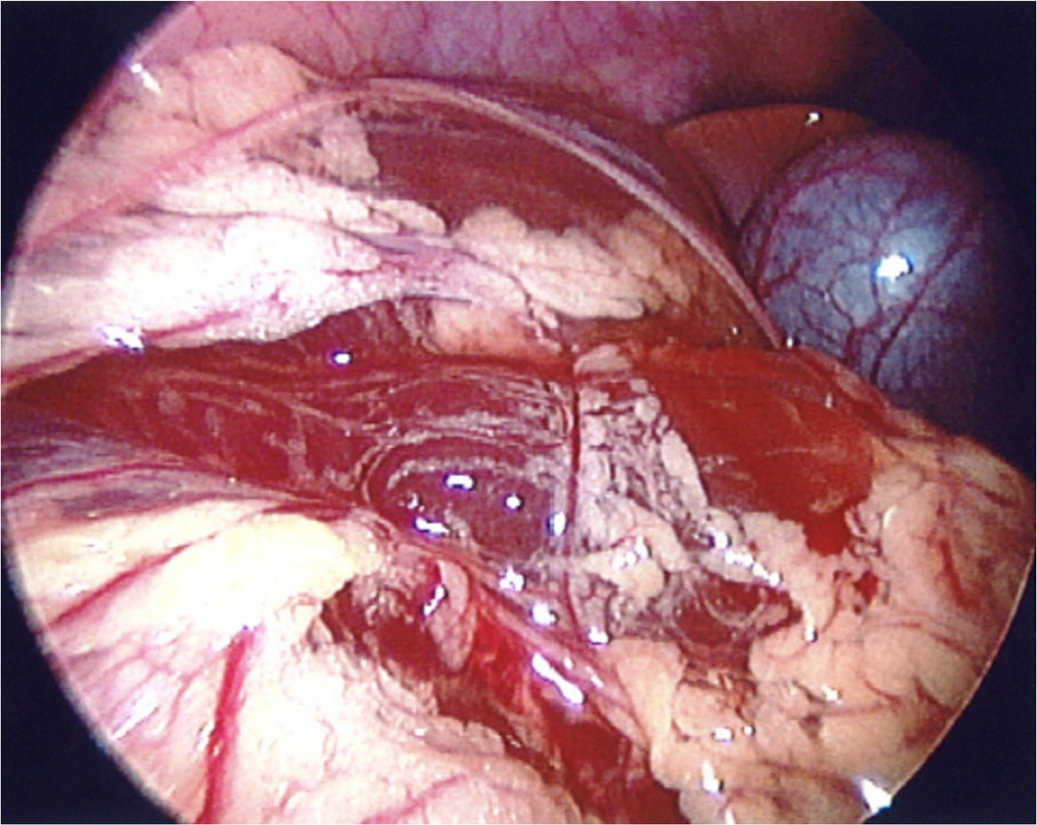
**Laparoscopy showing purulent multiloculated abdominal fluid and fibrinous membranes but no intestinal perforation.**

Laparoscopy did not reveal any intra-abdominal abnormality, especially no intestinal wall perforation, but the presence of abundant purulent abdominal fluid and fibrinous membranes in the lower abdomen, thus confirming the diagnosis of primary peritonitis. The appendix was removed. Histology showed no sign of acute appendicitis (Figure 1). The peritoneal fluid grew for a gram-positive, coccoid, aerobic, coagulase-negative, non-encapsulated germ, identified as a *Kocuria marina*. The organism was grown on BHI (brain heart infusion), a highly nutritious growth medium for fastidious and non-fastidious microorganisms. Blood culture was negative. Unfortunately, peritoneal fluid could not be sent for PCR. Blood PCR was negative for *K. marina*.

The patient was post-operatively admitted to the intensive care unit and treated with intravenous broad spectrum antibiotics (piperacillin and tazobactam) during 7 days. He gradually improved and was discharged on day 7, with a supplemental 7-day course of oral ciprofloxacin.

### Discussion

SBP is a rare presentation of peritonitis in childhood. Most cases are due to *Streptococcus pneumoniae*, *Escherichia coli* or staphylococcal strains [4]. However, in few cases no pathogen can be identified [5]. To our knowledge, this is both the first description of SBP caused by *Kocuria marina* in a child, and the first description of a *K. marina* SBP in a patient with no underlying risk factor.

Kocuria spp are members of the Micrococcaceae family. They are gram-positive, usually aerobic, coccoid, non-encapsulated bacteria. Seventeen species have been recognized so far [6]. They are environmental bacteria that can be encountered in mammals including humans’ skin and in the commensal flora of the oropharynx. Certain species (but not *K. marina*) have been isolated from traditional Korean fermented seafood [7]. Kocuria-related infections are rare. Bacteremia, peritonitis, brain abscess, endocarditis, cholecystitis and UTI have been described with *K. rosea*, *K. kristinae*, *K. varians*, and *K. rhizophila* (Table 1).

**Table 1:** **Reported cases of Kocuria spp infections in humans**

Species	Clinical presentation	Number of cases	Age (yrs)	Risk factor	Authors
*K. marina*	Peritonitis	1	3	None	Brändle, 2014
	Peritonitis	2	57, 73	PD	Lee, 2009
	Bacteremia	1	57	Cancer, CVC	Lai, 2011
*K. rhizophila*	Bacteremia	1	3	CVC	Moissenet, 2012
	Bacteremia	1	8	Methylmalonic aciduria	Becker, 2008
*K. varians*	Peritonitis	1	70	PD	Meletis, 2012
	Brain abscess	1	52	Diabetes	Tsai, 2010
*K. rosea*	Endocarditis	1	35	None	Srinivasa, 2013
	Bacteremia	1	60	HIV	Corti, 2012
	Bacteremia	1	39	Hematopoietic stem cell transplantation	Altuntas, 2004
	Peritonitis	1	8	PD	Dotis, 2012
	Peritonitis	1	56	PD	Kaya, 2009
*K. kristinae*	Bacteremia	3	2, 37, 68	Hypogammaglobulinemia, Cancer, CVC	Lai, 2011
	Endocarditis	1	89	IBD, post-operative, CVC	Lai, 2011
	UTI	1	20	Urinary catheter	Tewari, 2013
	Bacteremia	7	<1 yr	Preterm or leukemia, all CVC	Chen, 2013
	Bacteremia	1	68	Leukemia	Martinaud, 2008
	Bacteremia	1	51	Ovarian cancer	Basaglia, 2002
	Cholecystitis	1	56	None	Ma, 2005
	Endocarditis	1	74	Diabetes	Citro, 2013
	Peritonitis	1	69	PD	Cheung, 2011
	Peritonitis	1	78	PD	Carlini, 2011

*PD* peritoneal dialysis; *CVC* Central venous catheter; *HIV* Human immunodeficiency virus; *IBD* Inflammatory bowel disease; *UTI* Urinary tract infection.

*Kocuria marina* has been isolated from marine sediments and first identified in 2004 [8]. In 2009, Lee et al. described two cases of primary peritonitis caused by *K. marina* in adult patients undergoing peritoneal dialysis [9]. A small case series reported a positive *K. marina* blood culture in a 57 year-old oncologic patient with a central venous catheter [10]. However, it was considered a contaminant by the authors. We did not find any other report of *K. marina* infection.

In our patient, the organism was identified on peritoneal fluid samples, obtained at the time of surgery. Contamination of the fluid from the skin or from the intestinal content seems very unlikely because of the sterile skin condition at the time of surgery and the absence of visible intestinal perforation. The sample was seeded on the surface of a 5% sheep blood agar and incubated at 35°C in 5% CO2. After 48 h of incubation, the isolate was identified as Kocuria marina by MALDI-TOF mass spectrometry (score value = 2.014). MALDI target plates were inoculated by picking a freshly grown overnight colony with the tip of a sterile toothpick and smearing the specimen directly onto a ground steel MALDI target plate in a thin film. The microbial film was then overlaid with 1.5 μl of a MALDI matrix (a saturated solution of α-cyano-4-hydroxy-cinnamic acid in 50% acetonitrile–2.5% trifluoroacetic acid) and allowed to dry at room temperature. Mass spectra were acquired using the MALDI-TOF spectrometer in a linear positive mode (Microflex; Bruker Daltonics). Measured mass spectra ranged from 2,000 to 20,000 Da. Extraction of the peaks from the generated mass spectra and their matching against the reference spectra of the integrated database provided by the manufacturer was performed with MALDI Biotyper software (Bruker Daltonics). Unfortunately, the peritoneal fluid could not be sent for PCR-based confirmation of the species and antibiotic susceptibilities were not performed.

According to a 2010 review [6] which focuses on antibiotic susceptibilities for *Kocuria* species, *K. marina* should respond to ampicillin and other betalactam-based antibiotics while showing a relative resistance to penicillin [10]. At the time of presentation our patient had been treated with oral cephalosporin for 9 days, which might have covered early symptoms and might explain the acute severe clinical presentation.

Another interesting finding is the description of the presence of *K. marin*a in seafood and marine sediments. We can assume a potential relationship between the daily fish and seafood consumption over the 10-day period preceding the symptoms in our patient and the peritoneal infection due to this bacterium. Indeed, haematogenous dissemination is frequently described with Kocuria species. In our patient, the pathogen might have been ingested with sea-food, then translocated to mesenteric lymph nodes and to the peritoneal cavity. However, other pathophysiologic mechanisms are possible. Indeed, transient bacteremia following upper respiratory tract colonisation in the context of the upper respiratory tract infection preceding the peritonitis may also be encountered, even if less probable and not documented in this case.

Direct infection through cutaneous breaches associated with sea-bathing seems unlikely in our patient [11].

## Conclusion

The present report illustrates the first clinical presentation of *Kocuria marina* SBP in a child with no underlying risk factor for peritonitis. Although not previously described in children, this pathogen may be considered as a possible cause of SBP in a child.

## Consent

Written informed consent was obtained from the parents for publication of this case report and any accompanying images. A copy of the written consent is available for review by the Editor of this journal.

## Authors’ original submitted files for images

Below are the links to the authors’ original submitted files for images. Authors’ original file for figure 1
