# Supplementary figures and images for: First report of Kocuria marina spontaneous peritonitis in a child
Source: BMC Infect Dis. 2014 Dec 30;14:719. doi: 10.1186/s12879-014-0719-5 (PMC4297396; doi:10.1186/s12879-014-0719-5)

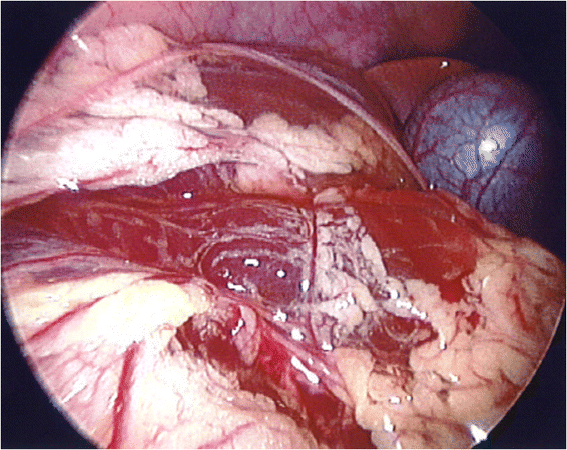

Supplement: Supplementary file 1 — Authors’ original file for figure 1 [file 12879_2014_719_MOESM1_ESM.gif]
